# Interactive effects of drought and edge exposure on old-growth forest understory species

**DOI:** 10.1007/s10980-022-01441-9

**Published:** 2022-05-20

**Authors:** Irena A. Koelemeijer, Johan Ehrlén, Mari Jönsson, Pieter De Frenne, Peter Berg, Jenny Andersson, Henrik Weibull, Kristoffer Hylander

**Affiliations:** 1 Department of Ecology, Environment and Plant Sciences, Stockholm University, 106 91 Stockholm, Sweden; 2 Bolin Centre for Climate Research, Stockholm University, 106 91 Stockholm, Sweden; 3 Swedish Species Information Centre, Swedish University of Agricultural Sciences, 750 07 Uppsala, Sweden; 4 Forest & Nature Lab, Department of Environment, Faculty of Bioscience Engineering, Ghent University, Geraardsbergsesteenweg 267, 9090 Melle-Gontrode, Belgium; 5 SMHI (Swedish Meteorological and Hydrological Institute), 601 76 Norrköping, Sweden; 6 Naturcentrum AB, Strandtorget 3, 444 30 Stenungsund, Sweden

**Keywords:** Edge effects, Extreme weather, Fragmentation, Land-use, Microclimate, Woodland key habitats

## Abstract

**Context:**

Both climatic extremes and land-use change constitute severe threats to biodiversity, but their interactive effects remain poorly understood. In forest ecosystems, the effects of climatic extremes can be exacerbated at forest edges.

**Objectives:**

We explored the hypothesis that an extreme summer drought reduced the richness and coverage of old-growth forest species, particularly in forest patches with high edge exposure.

**Methods:**

Using a high-resolution spatially explicit precipitation dataset, we could detect variability in drought intensity during the summer drought of 2018. We selected 60 old-growth boreal forest patches in central Sweden that differed in their level of drought intensity and amount of edge exposure. The year after the drought, we surveyed red-listed and old-growth forest indicator species of vascular plants, lichens and bryophytes. We assessed if species richness, composition, and coverage were related to drought intensity, edge exposure, and their interaction.

**Results:**

Species richness was negatively related to drought intensity in forest patches with a high edge exposure, but not in patches with less edge exposure. Patterns differed among organism groups and were strongest for cyanolichens, epiphytes associated with high-pH bark, and species occurring on convex substrates such as trees and logs.

**Conclusions:**

Our results show that the effects of an extreme climatic event on forest species can vary strongly across a landscape. Edge exposed old-growth forest patches are more at risk under extreme climatic events than those in continuous forests. This suggest that maintaining buffer zones around forest patches with high conservation values should be an important conservation measure.

**Supplementary information:**

The online version contains supplementary material available at 10.1007/s10980-022-01441-9.

## Introduction

Extreme weather events are increasing in frequency in many parts of the world (IPCC 2021) and may have larger effects on biodiversity than gradual climate change (Pecl et al. 2017; Maxwell et al. 2019). At the same time, land-use change and fragmentation are major drivers of biodiversity change (Foley et al. 2005; IPBES 2018). As both these drivers occur simultaneously, identifying their interacting effects on biodiversity is a key challenge in ecology and conservation (Mantyka-Pringle et al. 2012; Schulte et al. 2020). Extreme droughts and heatwaves are significant drivers of understory biodiversity change in forests (Archaux & Wolters 2006). Yet, the combined effects on biodiversity of such events and edge exposure due to forest fragmentation remains a knowledge gap (Williams & Newbold 2020). This is problematic, as a substantial part of earth’s forest biodiversity currently is confined to small and isolated remnants embedded in disturbed and intensively managed landscapes (Aune et al. 2005; Wintle et al. 2019).

Forest canopies buffer climate extremes, resulting in lower temperatures and higher humidity in the forest understory compared to open habitats during summers (Geiger 1965; De Frenne et al. 2021). In managed forest landscapes with recurrent clear-cut logging, the buffering capacity is constantly changing (Greiser et al. 2018), which ultimately influences the extent to which understory biodiversity is exposed to climatic variation. In Fennoscandian boreal forests, small and interspersed areas of old-growth forests (on which many species rely) have been delineated to conserve valuable biodiversity, and are referred to as *Woodland key habitats* (Nitare & Norén 1992; Timonen et al. 2010). However, they are frequently surrounded by clear-cuts and young replanted stands, creating large areas of forest edge habitat where high light radiation and wind speed increase desiccation and heat stress in the understory (Chen et al. 1993; Hylander 2005; Harper et al. 2015). Such harsher microclimate reduces the occurrence of some forest interior species (Moen and Jonsson 2003), and hamper biodiversity conservation (Aune et al. 2005). In conjunction with macroclimatic drought, the understory microclimate in edge habitats may become even more detrimental to sensitive organisms, and lead to rapid local extinctions of old-growth forest species. This could threaten understory biodiversity of small forest patches adjacent to clear-cuts.

It remains uncertain how different species groups respond to extreme events in managed forest landscapes, and advancing our knowledge of this field is crucial to adapt conservation measures to a changing climate. Especially old-growth forest species can be sensitive to changes in microclimatic conditions, as they are adapted to stable and humid conditions (Esseen 1997; Perhans et al. 2009), including many species of lichens, bryophytes and vascular plants. Due to the reduction in natural forests throughout the landscape, many old-growth forest species are of conservation concern, and occur in low abundances, making local extinction risks in small habitat patches high (Hanski 1998). Still, there is a large variation among species and species groups in their resilience to desiccation. Species on different substrates (soil, rocks, trees, logs) may experience different microclimates (Hylander et al. 2005; Davis et al. 2019) and species associated with shaded spruce forests may be more sensitive than species connected to open pine forests (Ranlund et al. 2018). The physiological adaptations to tolerate or resist drought differs largely between vascular plants, that regulate their internal water balance, and bryophytes and lichens (i.e. poikilohydric organisms), in which their internal water balance largely follows the environment (Green & Lange 1995; Esseen 1997). This could make poikilohydric organisms particularly sensitive to changes in environmental conditions (Perhans et al. 2009), but on the other hand, some species may survive longer periods in a desiccated state by simply shutting down their photosynthesis until rehydration (Green & Lange 1995). Many lichens can utilize water vapor and remain vital even in the absence of precipitation, whereas many bryophytes and lichens with cyanobacteria as photosymbiont require liquid water (Green & Lange 1995).

Here we examined the interactive effects of drought and microclimatic edge effects on old-growth forest understory species. We investigated the effects of the unusually severe drought in 2018. (Buras et al. 2020; Schuld et al. 2020). We used a novel spatiotemporally high-resolution precipitation dataset to differentiate between small woodland key habitats (1.5–3.0 ha) across central Sweden that had experienced different levels of drought intensity during this extreme summer (Berg et al. 2016). These sites also had different levels of edge exposure as a result of surrounding forest management (e.g. amount of recent clear-cuts). We surveyed old-growth forest indicator and red-listed species of bryophytes, lichens and vascular plants in 60 sites the year after the drought. We know that closely related species can die off shortly after drastic changes in microclimate, even within one growing season (Hylander et al. 2005; Dynesius et al. 2008). Since our study species grow slow and are most likely not visible directly after colonization, the size of their populations, as well as changes in species richness at the site level, would be visibly impacted the year after a severe drought. We hypothesized that the richness of the focal species in a forest patch would be lower, and the species composition different, after having been exposed to higher drought intensity. We hypothesized that the effects of drought intensity on the understory species community would increase with edge exposure of the forest patches. In other words, we expected interactive effects between drought and edge effects. We assessed these hypotheses for different subsets of the species, based on organism group and substrate association.

## Methods

### Study area

**Fig. 1:**
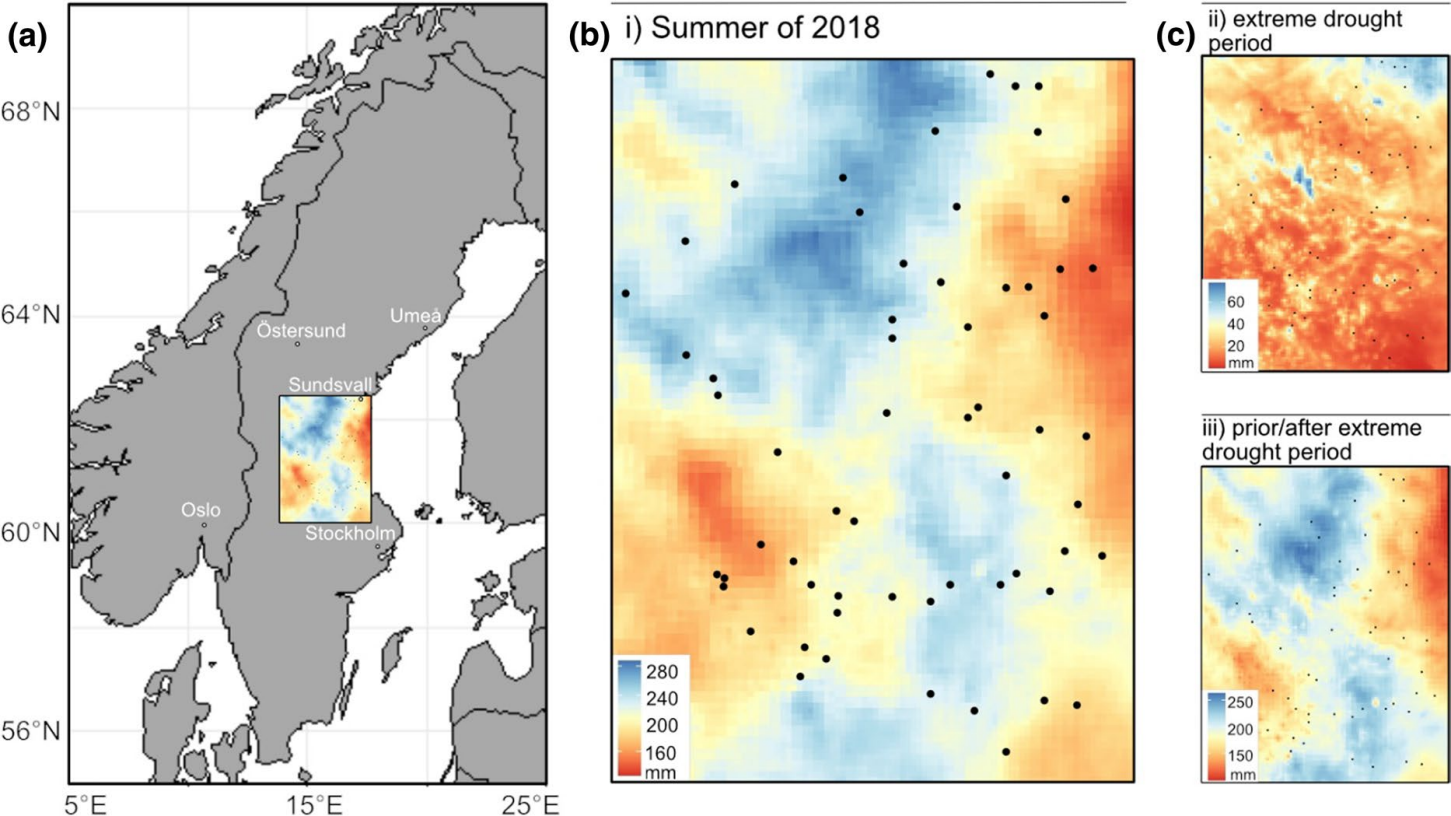
**a** The study area in central Sweden. **b**, **c** Maps showing the spatial variation of the drought indices based on HIPRAD data that we included in model 1 (b): (i) precipitation in mm during the summer 2018 (1st May–August 31st ) and model 2 (c): (ii) precipitation during the extreme drought period (22nd June–27th July 2018) and (iii) precipitation prior to and after this drought period (1st May–21st June and 28th July–31st August 2018). The black dots represent the 60 sites

The study was conducted in central Sweden, 13.5–17.2 °E longitude and 60.2–62.5 °N latitude, in the counties Dalarna, Gävleborg, Västernorrland, and Jämtland (Fig. 1a). The area has a cold temperate climate with distinct seasonality and a mean annual temperature of 3 °C. The average annual precipitation ranges between 600 and 800 mm per year, with highest precipitation during the summer (SMHI 2017). The summer of 2018 was classified as a climatic extreme and was one of the most severe droughts in the last 500 years in Europe (Schuld et al. 2020). Some places in central Sweden only received half of the average summer precipitation and experienced summer (JJA) temperature anomalies of + 2 to 3 °C (SMHI 2019).

The study area is characterized by a large cover of production forests, dominated by stands of *Picea abies* (L.) H.Karst and *Pinus sylvestris* L. (taxonomic source: dyntaxa.se throughout the paper). Only a small fraction of old-growth boreal forest remains as scattered small patches throughout the landscape, with large negative effects for many species (Aune et al. 2005). They have been carefully mapped because of their high conservational value and are denoted as *Woodland key habitats* (Nitare & Norén 1992; Johansson & Gustafsson 2001; Timonen et al. 2010).

We selected 60 woodland key habitats in central Sweden in a restricted area with similar land-use history and macroclimate, but at the same time displaying a large gradient in drought intensity during the summer of 2018 (Fig. 1). Some suffered an intense drought, whereas others received local, regular showers. The selection of the focal 60 sites was also based on information (available from the Swedish Forest Agency) about vegetation type, soil type, topography, form and size, to keep variation in these variables as low as possible. We selected woodland key habitats that were dominated by *P. abies* in the canopy (minimum 50% of the basal area, mean 79%), and had high cover of the dwarf shrub *Vaccinium myrtillus* L. and several pleurocarp moss species dominating in the understory vegetation. The sites ranged from 1.5 to 3.0 ha. We included sites with high edge exposure (adjacent to clear-cuts, young forest stands or fields), as well as sites embedded in more continuous forest structures.

### Climate & weather data

We calculated drought intensity for each site using the novel and high-resolution dataset on precipitation called HIPRAD (HIgh-resolution Precipitation from gauge-adjusted weather RADar, Berg et al. 2016, Van de Beek. in prep.) The dataset is based on high spatio-temporal resolution weather radar measurements (15 min; 2 km) that was additionally filtered to remove non-precipitation echoes recorded by the radar, and homogenized by adjustment at a 31-day running window to a coarser gridded station-based data set. For all sites, we calculated three measures that described different aspects of the drought (Fig. 1b): (i) total precipitation during the whole summer of 2018 (1st May–August 31st ); (ii) precipitation during the extreme drought period during the summer of 2018 (22nd June–27th July), when precipitation was nearly absent for 36 days in some areas of central Sweden; (iii) the summed precipitation before and after this extreme drought period in (ii) (1st May–21st June and 28th July–31st August).

To account for climatic gradients across the landscape that may have affected differences in the species pool, we included two background climatic variables. First, we calculated a measure of the average summer precipitation by averaging the cumulative rainfall from 1st May to 31st August over the years 2010 till 2017 (Online Appendix, Fig. S1a), using the HIPRAD dataset (2 km spatial resolution). Second, we included growing degree days (GDD, Fig. S1b), measured as days with mean temperatures over 5 °C, with a high (50 m) spatial resolution, to pick up variation in length of the growing season (Meineri & Hylander 2017).

### Species surveys and local environmental factors

We conducted surveys of red-listed and old-growth forest indicator species of bryophytes, lichens and vascular plants in the 60 sites between the end of June and mid-August 2019, the year after the drought. These species are closely affiliated with old-growth forest habitat and likely sensitive to habitat disturbances and associated (micro) climatic changes (Aune et al. 2005; Perhans et al. 2009). More specifically, we inventoried a subset of the species list from the monitoring program of woodland key habitats (Swedish Forest Agency, unpublished; Nitare & Norén 1992), that could occur in the woodland key habitats of our study region based on their ecology (resulting in a list of more than 100 species, of which 75 species were found, Online Appendix Table S2). We thoroughly inventoried the whole forest patch. We subdivided each site into 20 × 20 m subplots, with sometimes smaller subplots of different shapes at the edges due to the intersection between the overlaid grid and the woodland key habitat borders (when these intersection subplots were very small, they pooled together to obtain similar sizes to the other subplots). For each present species, we counted the total number of distinct occurrences of the focal species (i.e. occupied number of trees, logs, distinct patches) and estimated their total cover in each subplot (dm^2^). In the case of the orchid *Goodyera repens* (L.) R.Br., we also estimated the percentage of flowering individuals for the first encounter in each subplot, as a measure of the reproductive fitness of the population. We inventoried up to 2 m high for epiphytes. In each site, we identified and estimated the proportion of the tree species in the canopy layer, and estimated the number of downed logs as the number of dead trees > 30 cm diameter at breast height. Finally, we mapped the type of habitats (i.e. clear-cuts, young forests, fields etc.) that surrounded the focal site.

We categorized all surveyed species based on organism groups and substrate association (Online Appendix, Table S2). Organism groups were: lichens, bryophytes and vascular plants. Lichens were further divided into lichens with cyanobacteria as their main photobiont (cyanolichens) and lichens with green algae as main photosymbiont (chloro- and cephalolichens, hereafter chlorolichens). For substrate association we separated species as epiphytic (on trees), epixylic (on wood), epilithic (on stone) and epigeic (on soil). Epiphytes were further categorized based on their main host tree, since epiphyte communities vary depending on bark characteristics of host-tree species. We divided them into “high-pH bark” and “low-pH bark”, since pH is one of the major drivers of epiphytes, although bark structure also matters. Broadleaved trees such as *Populus tremula* L. and *Salix caprea* L., have a higher bark pH and harbor a more diverse epiphyte community than conifers (*P. abies* and *P. sylvestris*) and *Betula* spp. L., which have bark with a lower pH (Esseen et al. 1997).

### Edge effects

We categorized the strength of microclimatic edge effects for each subplot in each site as either strong, weak or absent, based on previous measures of how microclimatic edge effects penetrate edges with different edge orientation at forest/clear-cut interfaces as follows: We assumed strong edge effects for subplots adjacent (< 20 m) to south-facing clear-cuts or fields (including south–west and south–east facing). We assumed weak edge effects for the second row of subplots (20–40 m from the forest edge), as well as in subplots directly adjacent to north-facing edges (including north-west and north-east). Subplots bordering south facing edges adjacent to regenerating forest with young trees of > 2 m height were assumed to have weak microclimatic edge effects.

All other subplots were categorized as forest interior, where we assumed edge effects to be negligible.

For analyses at the subplot level, we used the three categories (strong edge effects, weak edge effects, forest interior). For the analyses at the site level, we used a continuous measure of edge exposure for each site, calculated as the proportion of subplot-area that were exposed to edge effects (including both weak and strong edge effects).

### Data analyses

**Fig. 2:**
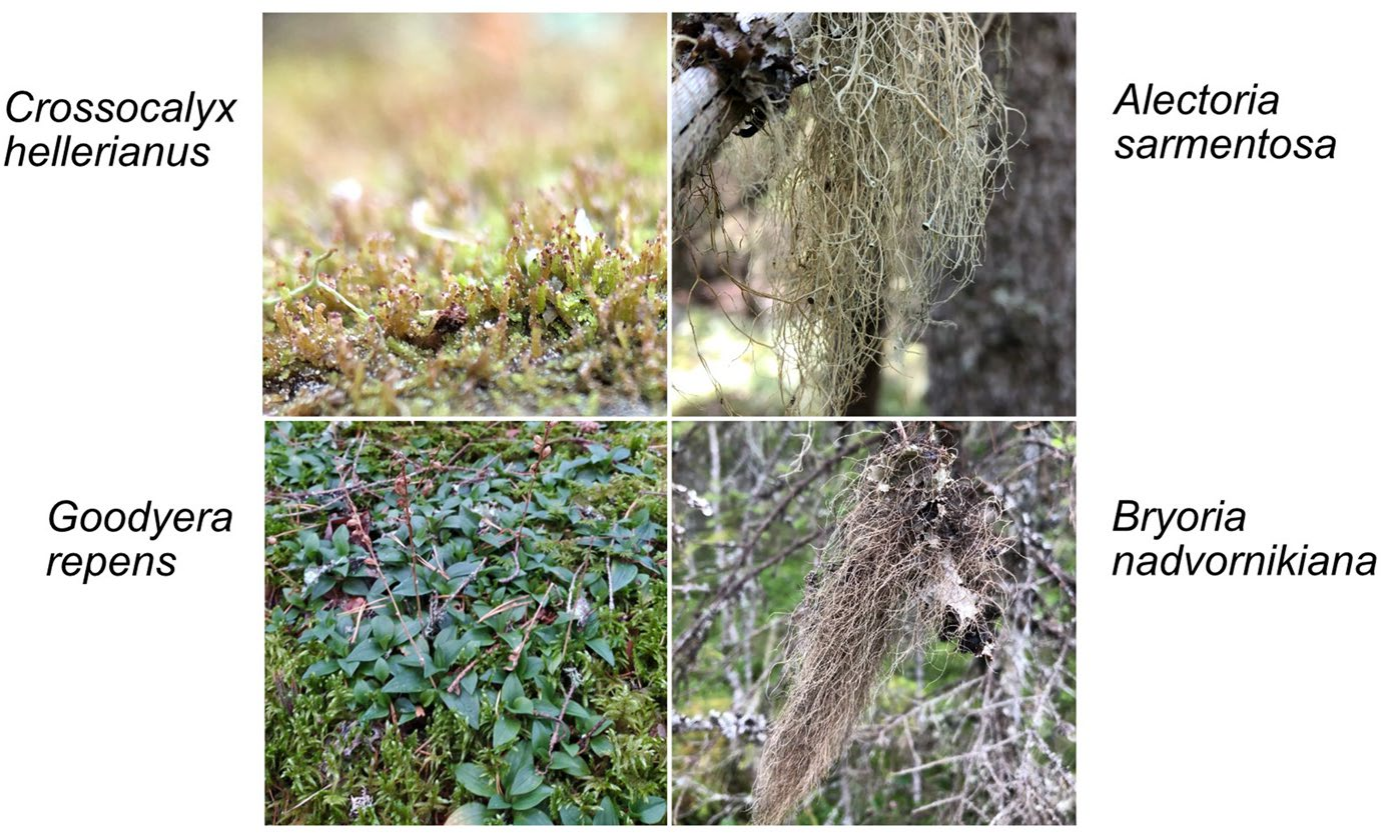
The four most commonly found species in our rare and indicator species surveys. Changes in cover in response to drought intensity and edge exposure were evaluated for these species

Analyses were conducted in R version 4.0.5 (R Core Team 2021). To examine if variation in drought intensity and edge exposure was related to the number and species composition of the red-listed and indicator species in the sites the year after the drought, we modeled these community descriptors as functions of drought intensity, edge exposure and their interaction. We created two models. In model 1 we included the total summer precipitation in 2018 (note that low precipitation corresponds to high drought intensity) as the measure of drought intensity (Fig. 1b). In model 2 we included precipitation during the extreme drought period, as well as precipitation prior and after this period as measures of drought intensity (Fig. 1c). In both models, we included average summer precipitation and growing degree days as co-variables to account for possible background geographical variation in the richness and composition of the species pool related to these climate gradients. We tested for an interaction between the background climate co-variables and drought intensity on species richness, but since it was not significant (p = 0.35), final models are presented without the interactions. We ran these models for overall species richness both at the site level and at the subplot level, as well as species richness in the different categories based on organism group and substrate association at the site level. We used generalized linear models at the site level and generalized mixed effects models at the subplot level within sites. We tested the models for species composition using a CCA (canonical correlation analysis). Finally, we tested the models for the distribution of the four most common species. These were the orchid *G. repens* (n = 42), the lichens *Alectoria sarmentosa* (Ach.) Ach. (n = 39) and *Bryoria nadvornikiana* (Gyeln.) Brodo & Hawksw. (n = 32), and the bryophyte *Crossocalyx hellerianus* (Nees ex Lindenb.) Meyl. (n = 34) (Fig. 2). We did this both at the site level using linear models and at the subplot level using linear mixed effect models. We modeled the total cover of the species (in dm^2^) at the site level and the proportional coverage (in dm^2^/m^2^) at the subplot level for the different categories of edge effects. For models at the site level, we used a continuous measure of edge exposure, while at the subplot level within sites edge exposure was a three-level ordinal factor with the previously defined strong edge effects, weak edge effects and forest interior. All explanatory variables were scaled in order to obtain comparable standardized coefficients. Information about model structure, additional covariates, and model diagnostics are detailed in the supplementary information (Online Appendix, Supplementary Methods).

## Results

### Explanatory variables

Rainfall during the summer of 2018 ranged from 144 to 265 mm across the different sites, resulting in anomalies within sites between 47 and 82% of the average summer precipitation during the period 2010–2017 (Online Appendix, Table S3a). This indicates that some sites experienced the majority of regular rainfall, whereas others experienced more intense drought. Precipitation during the extreme drought period in July ranged from 4 to 56 mm. Some sites suffered 36 days of almost no rainfall whereas others received one or several (even heavy, of 20 mm) showers in this period. Edge exposure in the sites ranged from 0 to 62% (mean 15%), and 42 sites had some level of edge exposure.

### Species richness

**Fig. 3:**
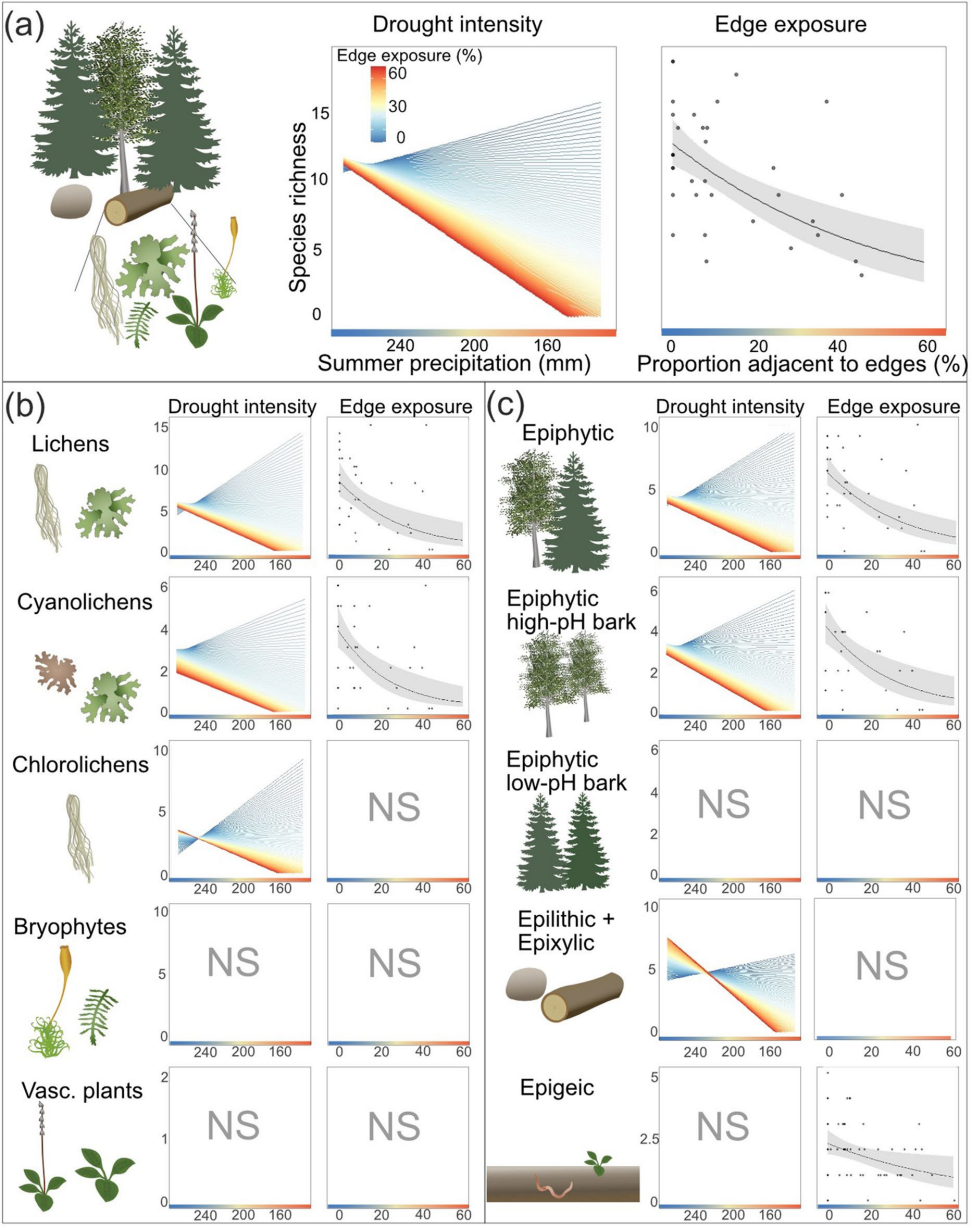
The relationship between old-growth forest species richness at the site-level and drought intensity, edge exposure and their interaction in model 1 (i.e. with drought intensity being the total summer precipitation in 2018). Species richness was analyzed in total (**a**), and categorized into organism group (**b**) and substrate association (**c**). We show the predicted patterns, and in case of edge exposure also the raw data, for the significant relationships. High uncertainty around the prediction in the interaction plots is indicated by lines diverging from each other (e.g. blue lines going in both positive and negative directions for low edge exposure) and low uncertainty by lines clustering close together into the same direction (e.g. for high edge exposure, red lines). Thus, the graph in panel “a”, for instance, should not be interpreted as if there are positive effects of drought for sites with low edge exposure. Non-significant relationships are indicated by NS. The corresponding statistics (standardized parameter estimates and significance) can be found in Table 1

The number of focal red-listed and old-growth forest indicator species in the sites ranged from 3 to 20, with a mean of 9.4. Species richness was negatively related to lower summer precipitation in combination with a high edge exposure (i.e. there was a significant interaction effect, Table 1; Fig. 3a). Models predicted that forest patches with an edge exposure of more than 30% were affected by drought intensity, i.e. a predicted negative relationship between species richness and drought intensity at the high end of the edge exposure gradient (orange–red lines, Fig. 3a, b, c). Forest patches with lower edge exposure were not affected (the predicted relationships, blue lines, in Fig. 3a, b, c show no clear relationship). We found the strongest effects on cyanolichens and epiphytic species on trees with high-pH bark, and for the pooled group of epilithic and epixylic species (Table 1; Fig. 3b, c). In contrast, species richness of vascular plants and epiphytes on trees with low-pH bark were not related to drought intensity, edge exposure, or their interaction (Table 1; Fig. 3b, c). Chlorolichens were weakly associated with combined effects of drought and edge exposure, bryophytes weakly with drought intensity, and epigeic organisms with edge exposure, but with negligible pseudo R^2^-values and thus high uncertainty.

**Table 1:** The relationship between old-growth forest species richness at the site-level and drought severity, edge exposure and their interaction in model 1. Drought intensity is defined as the absolute precipitation (low precipitation is high drought intensity), in this model over the whole summer of 2018, 1st May - August 31st. Negative coefficients thus denote a negative relationship between drought and species richness. Species richness was analyzed in total, and categorized into organism group and substrate association. The table shows the standardized parameter estimates and significance is indicated as follows: *** p < 0.001 ** p < 0.01, * p < 0.05, . p < 0.1. Interaction effects are best evaluated by inspecting the graphs in Fig. 3

	Drought intensity	Edge exposure	Interaction	Background climate		Pseudo R^2^ main variables	Total num of species
	Summer 2018	Edge exposure (%)	Summer drought *Edge exposure	Average summer precipitation (mm)	Growing degree days		
Overall^a, P^	− 0.09	− 0.32 ***	− 0.19 **	− 0.09	− 0.33***	0.002	75
Organism group							
Lichens^a, QP^	− 0.07	− 0.55**	− 0.31 **	− 0.15	− 0.61***	0.05	39
Cyano lichens^a, P^	− 0.18	− 0.70***	− 0.33*	− 0.20	− 0.51**	0.13	13
Chloro lichens^a, QP^	− 0.02	− 0.45	− 0.31*	− 0.11	− 0.70**	0	26
Bryophytes^a, P^	− 0.25*	− 0.09	− 0.06	− 0.08	− 0.08	0.08	29
Vascular plants^b, QP^	0.11	− 0.18	− 0.11	− 0.07	0.10	0	7
Substrate association							
Epiphytic^a, P^	− 0.04	− 0.47***	− 0.25**	− 0.18.	− 0.50***	0	31
Epiphytic low-pH bark^b,P^	0.15	− 0.08	− 0.04	− 0.18	− 0.50 ***	0	12
Epiphytic high-pH bark^a,P^	− 0.13	− 0.54**	− 0.30**	− 0.23	− 0.42**	0.10	19
Epilitic + epixylic^b, QP^	− 0.11	− 0.20	− 0.22**	− 0.14	− 0.26	0.20	42
Epigeic^b, QP^	− 0.07	− 0.24*	− 0.06	− 0.09	− 0.07	0	14

Drought intensity is defined as the absolute precipitation (low precipitation is high drought intensity), in this model over the whole summer of 2018, 1st May–August 31st. Negative coefficients thus denote a negative relationship between drought and species richness. Species richness was analyzed in total, and categorized into organism group and substrate association. The table shows the standardized parameter estimates and significance is indicated as follows: ***p < 0.001, **p < 0.01, *p < 0.05, p < 0.1. Interaction effects are best evaluated by inspecting the graphs in Fig. 3

*P* Poisson distribution, *QP* quasi-Poisson distribution

^a^n = 35

^b^n = 60

Similar results were obtained for models where we separated the precipitation over the whole summer into the extreme drought period and the period prior and after this period, (Fig. 1c, Online Appendix, Table S4). Precipitation prior and after the extreme drought, in interaction with edge exposure, was strongly associated with species richness of the above-mentioned groups.

We did not find significant effects of any drought intensity measure, edge exposure nor their interaction on species richness at the subplot level (Online Appendix, Table S5).

### Species composition

**Fig. 4:**
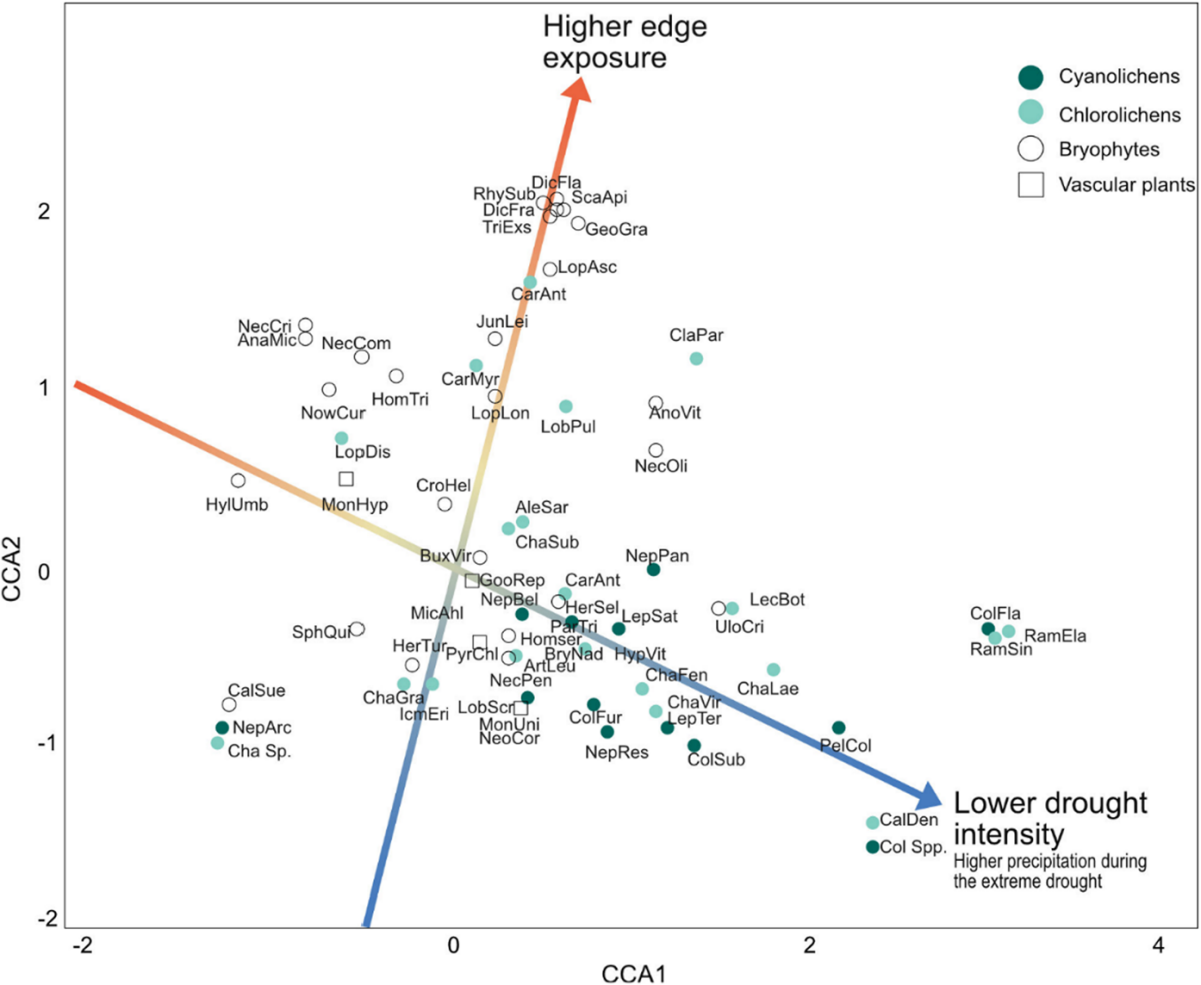
The association between old-growth forest species composition and drought severity and edge effects at the site level, showing the CCA species scores and the significant explanatory variables (p < 0.05) after accounting for background climatic variables. The different colors and shapes represent the different organism groups. Species acronyms are based on the first three letters of the genus part and the first three letters of the species part of their scientific names, for example *Goodyera repens* = GooRep (full names can be found in Online Appendix Table S1). The eigenvalues were 0.37 for axis 1 and 0.30 for axis 2, and the inertia of the constrained (drought and edge exposure) and conditional (background climate) and variables were 0.12 and 0.14 respectively

The composition of the old-growth forest indicator species at the site level was not associated with the total summer precipitation in 2018, but was correlated with rainfall during the extreme drought (X^2^_1_ = 0.37, p = 0.005) and with edge exposure (X^2^_1_ = 0.30, p = 0.02). Furthermore, we found a trend-significant interaction between edge exposure and this drought intensity measure (X^2^_1_ = 0.33, p = 0.06). When plotting the CCA scores of the species, lichens seemed to be more associated with lower drought intensity and lower edge exposure than bryophytes (Fig. 4) and epiphytes on high-pH bark seemed more associated with these variables than species on other substrates (Fig. S5), which corresponds to the linear models on species richness of the different groups.

### Analyses of individual species

**Fig. 5:**
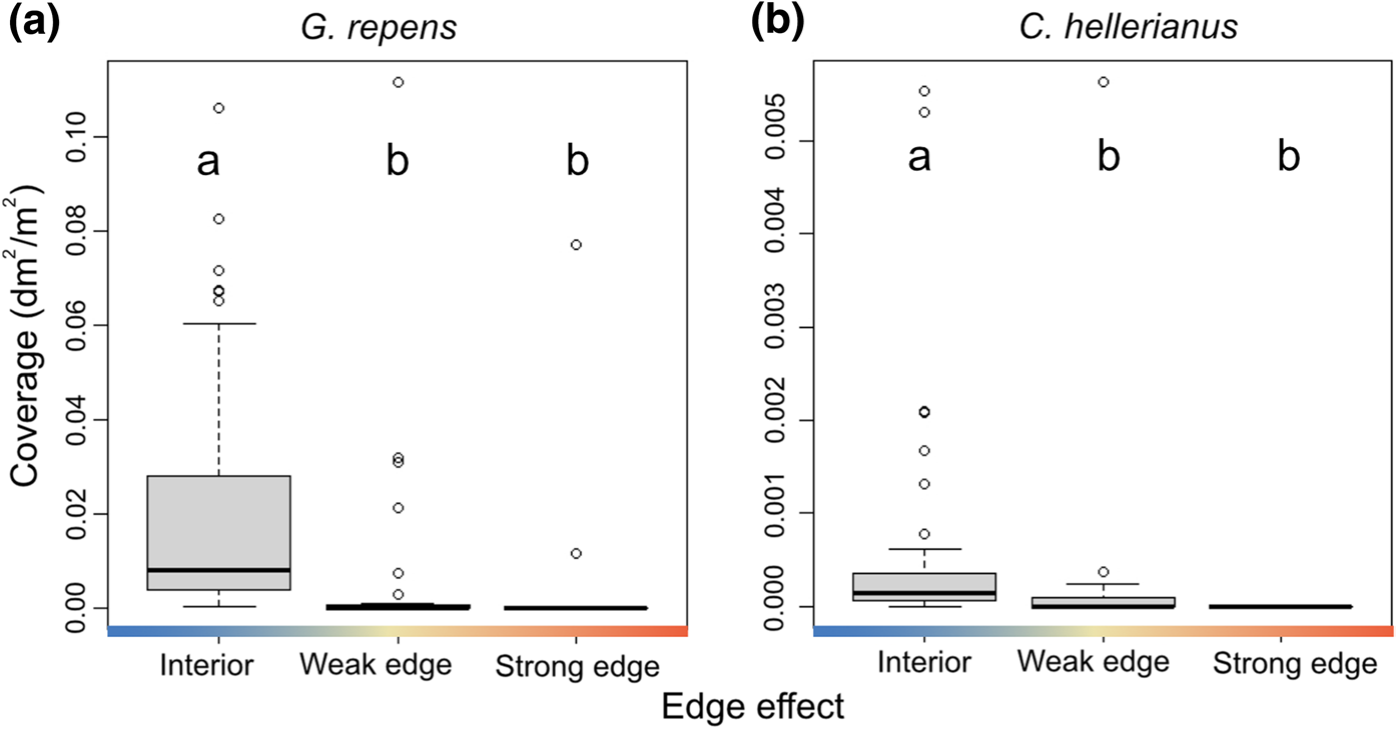
Coverage of the orchid *Goodyera repens* (**a**) and the bryophyte *Crossocalyx hellerianus* (**b**) for different levels of edge exposure at the subplot level within woodland key habitats. Different letters above the boxplots (**a** and **b**) indicate statistical differences between the edge effects. We removed one outlier in the interior for both species in these plots to improve visibility (plots with the total data are shown in Online Appendix, Fig. S2). Statistics corresponding to this analysis can be found in Online Appendix, Table S6

Neither drought intensity, edge exposure nor their interaction explained the presence or coverage of the four most common species when analyzed at site-level, nor the proportion of fertile individuals of *G. repens*. The distribution at the subplot level of the most common species was not explained by drought intensity, nor by the interaction between edge exposure and drought. However, *C. hellerianus* and *G. repens* were found in higher coverage, and with higher fecundity in case of *G. repens*, in the forest interior compared to subplots towards the forest edge (Fig. 5, Online Appendix, Table S6 and Fig. S4) and also *A. sarmentosa* was found in slightly higher densities in the forest interior compared to in sub-plots with strong edge influence (Online Appendix, Table S6).

## Discussion

Changes in climate and land-use are simultaneously threatening biodiversity, but their interactive effects are poorly understood. We found interactive effects of extreme summer drought intensity and edge exposure on old-growth forest species richness and species composition one year after the drought event. In other words, the risk of being negatively affected by an extreme drought seems to depend on where in the landscape a species occurs, and fragmentation makes biodiversity more exposed to such events. This has important implications for conservation strategies and landscape planning, and suggests that more continuous forest cover and buffers at exposed edges are important measures. Interestingly, not all species in our study were affected the same way, but cyanolichens, epiphytes on broadleaved trees and species on convex substrates (epixylic and epilithic) seemed to be driving the patterns.

Microclimatic edge effects are well known to impede conservation in forest ecosystems. Not least for conservation approaches that rely on small and interspersed patches across managed landscapes, such as delineating and protecting woodland key habitats (Aune et al. 2005). Also in our study, we found strong negative edge effects from clear-cuts and other open areas adjacent to the forest patches on sensitive old-growth forest indicator and red-listed species. Interestingly, our results suggest that species in edge exposed forest patches were impacted by drought, while more continuous forest cover seemed to have buffered the effects of the drought. Desiccation and heating of the understory, caused by the combination of drought and edge exposure, likely reduced abundances and led to local extinctions of the study species, which are rare and adapted to stable and humid conditions of old growth forests (Esseen et al. 1997; Aune et al. 2005; Löbel et al. 2012). The exact mechanisms that negatively affect understory organisms at forest edges during an intense drought are difficult to determine, but both wind and solar radiation increase towards edges and could interact with the summer drought, to create hostile conditions. The large temperature anomalies during the summer–drought have most likely further amplified harsh microclimates at the forest edge, but were not separately considered in our study due to lack of high-resolution data. Furthermore, solar radiation can directly exacerbate desiccation stress (e.g. for lichens, Gauslaa et al. 2012). However, to what extent the depth of edge influence (DEI) and the magnitude of edge influence (MEI) increase under extreme drought conditions remains unknown, and we could not analyze either those aspects on our response variables. Yet, since drought reduces evapotranspiration and thereby hampers the cooling of the understory (Davis et al. 2019), is it likely that the edge effect will be both more severe and penetrate deeper in dry years. In fact, when forest patches are small with exposed edges, they lose their buffering capacity even under normal weather conditions (Aune et al. 2005). Additionally, other factors than microclimate could have influenced edge habitat biodiversity, such as disturbance from adjacent land-use (Harper et al. 2015). Similar to microclimatic edge effects, disturbed canopy structure is likely to reduce the buffering of climate extremes in the understory (De Frenne et al. 2021). Such changes can, besides active management, occur as a result of drought-induced forest fires, increased insect outbreaks, or dieback of trees, particularly at forest edges (Buras et al. 2018), thereby further reducing the buffering capacity of forests against future droughts.

Patterns differed among organism groups and were strongest for cyanolichens and epiphytes on broadleaved trees, as well as species occurring on convex substrates such as trees and logs. Habitat requirements might be particularly restrictive for cyanolichens, which depend on liquid water to be metabolically active (Green and Lange 1995; Perhans et al. 2009). Furthermore, the majority of cyanolichens in our study were epiphytes on trees with high-pH bark (broadleaved trees such as *P. tremula* and *S. caprea*) (Online Appendix, Table S2). Since these trees occurred in relative low abundances in our study area, their epiphytes may be more likely to face local extinction (Hanski 1998). Contrary to our results, Ranlund et al. (2018) found that lichens (in general, not restricted to indicator and red-list species) on the conifer species *P. abies* were more sensitive to microclimatic changes compared to species on the broadleaved *P. tremula*, due to adaptation to more shaded microclimates in *P. abies* forests. Epiphytes on trees may experience harsher microclimatic conditions, since buffering of temperature and drought is reduced with height above the forest floor (Davis et al. 2019). Similarly, species affiliated to logs and rocks may have experienced increased desiccation due to the convex shape of their substrates, which could explain their negative correlation with drought in edge exposed patches. Similar patterns have been found for bryophytes on convex, compared to concave, substrates in small retained buffer strips (Hylander et al. 2005).

We found no or minimal associations of species richness with drought intensity and edge exposure for the other studied groups (chlorolichens, bryophytes, vascular plants, epiphytes on low-pH bark, and epigeous species). We do not know to what extent these species are not as sensitive to desiccation as commonly thought or have mechanisms to deal with a drought of this time-span of a few weeks. Physiological, morphological, ecological, demographic and life-history traits are all important factors of resilience, including resistance, tolerance and recovery to drought (Archaux & Wolters 2006). The difference between cyanolichens (strongly correlated with drought and edge exposure) and bryophytes (not correlated) was particularly surprising, since both are poikilohydric organisms depending on liquid water and since bryophytes have previously been shown to be sensitive to microclimatic changes (for example near clear-cuts, Esseen et al. 1997; Hylander et al. 2005). However, even if poikilohydric organisms are directly influenced by drought, they can revive after dehydration, and the duration and severity of the 2018 drought could allow recovery for some bryophyte and chlorolichen species. Chlorolichens have several adaptations to withstand harsh conditions, perhaps explaining the lack of negative edge influence on this group. First, they can use air humidity to sustain their metabolism and remain vital even in absence of precipitation. Second, they can adjust thallus thickness to light-intensity levels and thrive under more exposed conditions (Gauslaa et al. 2007; Perhans et al. 2009). However, there may be an interaction between drought and edge exposure on chlorolichens (significant effect but low R^2^), indicating a potential future trade-off in conservation strategies between management for optimal light conditions versus for increasing buffering capacity. A species’ microhabitat, e.g. if they occur on convex substrates or in concave protected sites such as crevices, could determine how exposed they were to drought events or edge effects (Hylander et al. 2005). Generally, species that occur in large abundances have a lower chance of going locally extinct. In line with this, our analyses of species at the subplot level, driven by the relatively more frequent species in our data set, showed no correlation with drought and edge effects, as opposed to the analysis of species richness between sites (driven by rare species). Similarly, species on common substrates, e.g. coniferous trees and *Betula* spp., could be more likely to persist (and in the long term, recover) due to greater habitat availability. Species on soil (mainly vascular plants) were negatively associated with edge exposure, but not with drought intensity. This could indicate that they are sensitive to (micro)climatic conditions, but that soil moisture has been sufficient to sustain populations during the drought. However, sample sizes of vascular plants and epigeic organisms were too small to draw any firm conclusions. In fact, several other traits that may affect resistance and tolerance to drought, but could not be differentiated in our analyses due to too low sample sizes. For example, morphology and reproductive systems determine responses to changes in climate (Löbel et al. 2018) and liverworts may be more sensitive than mosses (Hylander et al. 2005; Perhans et al. 2009; but see Löbel et al. 2012). We deem recovery of detectable populations within 1 year after the drought unlikely, even if the species were re-introduced, because many of our focal species are slow-growing. Moreover, re-introduction or colonization of old-growth specialist species is improbable in a landscapes mosaic of predominantly managed forests.

By definition, it is difficult to study the effects of rare and stochastic events, which is a major reason for our lack in understanding the effects of climatic extremes. The unavailability of data pre-dating rare events is often a great limitation that reduces the capacity to identify causal relationships. Researchers are in such instances left to other approaches. We had access to a novel high-resolution precipitation dataset that allowed us to differentiate between sites that had experienced different levels of drought intensity as a way of tackling this urgent research question. We kept covariation of potentially confounding factors as small as possible across the forest patches, and accounted for background climate in the statistical models. Still, we cannot rule out that differences in unmeasured factors (e.g. in climate, soil, substrate availability, land-use history) could have obscured existing patterns, or that correlations between such unmeasured factors and summer drought in 2018 potentially biased the results. Given the potential impact of unmeasured factors and stochasticity in occurrence patterns of rare species, we did not expect high R^2^-values in our models. Notwithstanding the above-mentioned limitations and the low R^2^ values, the fact that we found statistically significant patterns in this study is very intriguing and suggests that drought, combined with edge exposure, can influence population dynamics of sensitive understory species. Even if we cannot be certain that not recording a species in our survey represented a true absence, undetectability due to extremely low abundances after the drought reflects a poor state of the population and is ecologically relevant. Given the fast declines of many forest species close to newly formed clear-cut edges (e.g. Hylander et al. 2005; Dynesius et al. 2008), it is not surprising that an additional stressor, in our case a strong drought event, can lead to local extinctions of sensitive species that occur in small numbers under baseline conditions.

### Implications for conservation and management

Our study provides support for negative synergistic impacts of climatic extremes and edge exposure on species of conservation concern, which has important implications for landscape management. Conservation approaches in small and interspersed forest patches, such as woodland key habitats, may be less effective in a changing climate where a higher frequency and intensity of droughts and heatwaves are expected. This is important, since small and isolated patches play a critical role in conserving much of the world’s biodiversity (Wintle et al. 2019; Valdés et al. 2020). However, old-growth forest species seemed less affected by drought in patches embedded in more continuous forest structures, where humidity and temperature extremes were probably more effectively buffered (Davis et al. 2019; De Frenne et al. 2021). This indicates that forest management can be optimized to buffer the effects of climatic extremes by reducing the amount of edge habitat in the landscape. Relevant measures include to leave buffer zones surrounding old growth forest patches, and to adopt continuous-cover forestry instead of the currently dominant use of clear-cutting in this study area as a forest management strategy (Lundmark et al. 2013).

## Supplementary Information

Below is the link to the electronic supplementary material.

Supplementary material 1 (DOCX 697.2 kb)

## Data Availability

Data are available from the Dryad Digital Repository 10.5061/dryad.gtht76hpm (Koelemeijer et al. 2022).
