# Supplementary material for: Interactive effects of drought and edge exposure on old-growth forest understory species
Source: Landsc Ecol. 2022 May 20;37(7):1839–53. doi: 10.1007/s10980-022-01441-9 (PMC9250463; doi:10.1007/s10980-022-01441-9)
Supplement: Supplementary file 1 — Supplementary material 1 (DOCX 697.2 kb) [file 10980_2022_1441_MOESM1_ESM.docx]

**Supplementary Tables**

Table S1 Information on the 60 woodland key habitats, showing their coordinates, size, edge exposure, presence of broad-leaved tree species, proportion of *P. abies*, the drought severity and background climate variables and their total species richness.

|  |  |  |  |  |  |  | **Drought severity indices (2018)** | | | **Background climate** | |  |
| --- | --- | --- | --- | --- | --- | --- | --- | --- | --- | --- | --- | --- |
| **Woodland key habitat ID** | **Latitude (°N)** | **Longitude (°E)** | **Size (ha)** | **Edge exposure (%)** | **Broad leaved tree sp. present** | **Proportion *P. abies* (%)** | **Total summer precipitation (mm)** | **Distinct drought period (mm)** | **Prior/after the distinct drought (mm)** | **Average summer precipitation**  **(mm)** | **GDD (days over 10 years)** | **Species richness (of our list)** |
| N 280-2017 | 60,37 | 16,73 | 2,2 | 34,62 | 1 | 65 | 202,69 | 3,77 | 198,92 | 268,14 | 1537 | 6 |
| N 3050-1997 | 60,35 | 16,94 | 2,7 | 0,00 | 1 | 70 | 183,73 | 6,54 | 177,19 | 261,98 | 1548 | 11 |
| N 49-2004 | 60,71 | 16,81 | 1,7 | 27,05 |  | 70 | 219,31 | 20,90 | 198,41 | 298,43 | 1431 | 4 |
| N 8098-1994 | 60,77 | 16,58 | 3,0 | 7,80 | 1 | 75 | 226,88 | 44,23 | 182,64 | 296,63 | 1404 | 10 |
| N 793-2004 | 60,75 | 16,49 | 1,9 | 9,23 | 1 | 65 | 239,97 | 30,27 | 209,70 | 300,78 | 1353 | 9 |
| N 1362-2004 | 60,82 | 17,19 | 2,0 | 0,00 | 1 | 80 | 191,55 | 14,17 | 177,38 | 284,37 | 1529 | 6 |
| N 3492-1997 | 60,83 | 16,94 | 1,8 | 0,00 | 1 | 70 | 207,47 | 18,99 | 188,48 | 304,96 | 1468 | 12 |
| N 9506-1994 | 60,97 | 17,04 | 1,9 | 0,00 | 1 | 75 | 187,31 | 16,80 | 170,51 | 296,39 | 1486 | 9 |
| N 1607-1999 | 61,20 | 17,11 | 2,3 | 8,05 | 1 | 80 | 178,62 | 16,17 | 162,45 | 292,96 | 1481 | 4 |
| N 11346-1996 | 61,08 | 16,56 | 1,5 | 7,82 | 1 | 75 | 225,84 | 14,42 | 211,42 | 323,69 | 1273 | 13 |
| N 1625-2005 | 61,22 | 16,80 | 1,7 | 0,00 | 1 | 80 | 197,04 | 26,35 | 170,68 | 322,92 | 1376 | 15 |
| N 954-2007 | 61,27 | 16,29 | 1,6 | 0,00 | 1 | 60 | 193,31 | 20,18 | 173,13 | 293,36 | 1349 | 12 |
| N 471-2000 | 61,31 | 16,39 | 1,7 | 9,37 |  | 70 | 188,97 | 32,66 | 156,30 | 283,31 | 1374 | 4 |
| N 598-2013 | 61,56 | 16,33 | 2,0 | 7,67 | 1 | 95 | 196,74 | 16,14 | 180,59 | 311,63 | 1160 | 6 |
| N 8595-1998 | 61,57 | 16,86 | 1,8 | 0,00 | 1 | 80 | 171,23 | 19,64 | 151,59 | 334,44 | 1360 | 12 |
| N 2657-2001 | 61,73 | 17,22 | 1,5 | 0,00 | 1 | 85 | 143,48 | 22,64 | 120,84 | 276,46 | 1443 | 11 |
| N 65-2016 | 61,94 | 16,28 | 1,6 | 0,00 | 1 | 80 | 226,81 | 24,09 | 202,72 | 306,20 | 1193 | 19 |
| N 8816-1998 | 61,76 | 15,88 | 1,9 | 0,00 |  | 100 | 235,25 | 12,09 | 223,16 | 320,96 | 1232 | 7 |
| N 4550-1993 | 61,71 | 16,16 | 2,0 | 0,00 |  | 95 | 212,26 | 17,31 | 194,95 | 305,67 | 1272 | 6 |
| N 1137-2008 | 61,68 | 16,62 | 1,6 | 25,31 | 1 | 90 | 182,70 | 20,73 | 161,98 | 316,31 | 1176 | 9 |
| N 2080-2004 | 61,68 | 16,75 | 2,2 | 33,40 | 1 | 80 | 173,41 | 20,22 | 153,19 | 329,02 | 1291 | 7 |
| N 681-2017 | 61,73 | 16,99 | 1,6 | 0,00 |  | 70 | 152,41 | 20,45 | 131,97 | 296,11 | 1425 | 3 |
| N 1985-1995 | 61,94 | 17,07 | 1,6 | 4,53 | 1 | 70 | 177,53 | 37,35 | 140,18 | 305,40 | 1364 | 10 |
| N 6440-1995 | 62,16 | 16,89 | 1,6 | 7,23 | 1 | 80 | 211,25 | 43,56 | 167,69 | 311,08 | 1170 | 14 |
| N 1095-2003 | 62,30 | 16,92 | 1,5 | 1,37 | 1 | 60 | 217,84 | 55,82 | 162,02 | 305,54 | 1181 | 14 |
| N 3217-2003 | 62,32 | 16,76 | 1,9 | 15,18 | 1 | 55 | 218,48 | 54,93 | 163,55 | 301,78 | 1213 | 18 |
| N 4253-1997 | 62,35 | 16,57 | 2,2 | 5,13 | 1 | 80 | 219,69 | 51,46 | 168,23 | 293,80 | 1148 | 15 |
| N 1090-2008 | 62,18 | 16,16 | 2,2 | 42,07 | 1 | 75 | 247,45 | 30,23 | 217,22 | 311,09 | 1137 | 20 |
| N 850-2007 | 62,05 | 15,48 | 1,5 | 40,42 | 1 | 50 | 265,24 | 23,03 | 242,21 | 333,87 | 1159 | 9 |
| N 3309-2001 | 61,93 | 15,59 | 1,5 | 36,64 | 1 | 95 | 263,05 | 17,79 | 245,26 | 321,09 | 1284 | 16 |
| N 772-2001 | 62,04 | 14,71 | 1,9 | 28,16 | 1 | 70 | 224,41 | 16,07 | 208,34 | 390,75 | 1150 | 5 |
| N 10924-1997 | 61,85 | 14,35 | 1,9 | 30,73 |  | 80 | 234,72 | 37,48 | 197,25 | 409,88 | 955 | 13 |
| N 7865-1998 | 61,68 | 13,93 | 2,8 | 0,00 |  | 75 | 245,78 | 36,55 | 209,24 | 448,09 | 814 | 9 |
| N 9851-1995 | 61,42 | 14,53 | 2,1 | 0,00 | 1 | 75 | 235,93 | 29,80 | 206,12 | 440,26 | 1023 | 19 |
| N 3132-1993 | 61,49 | 14,37 | 2,0 | 16,91 |  | 90 | 250,96 | 31,51 | 219,45 | 443,17 | 995 | 7 |
| N 5413-1994 | 61,36 | 14,57 | 1,7 | 0,00 |  | 95 | 219,32 | 22,64 | 196,67 | 422,99 | 1013 | 9 |
| N 54-1996 | 61,17 | 14,99 | 1,6 | 8,10 |  | 90 | 200,75 | 8,96 | 191,79 | 362,49 | 1269 | 8 |
| N 1325-2009 | 60,76 | 14,59 | 1,5 | 24,34 |  | 90 | 153,95^*^ | 10,53^1^ | 143,42 | 359,47 | 1143 | 7 |
| N 1291-2009 | 60,79 | 14,62 | 2,0 | 8,32 | 1 | 80 | 152,60^*^ | 11,98^1^ | 140,61 | 359,47 | 1282 | 14 |
| N 42-2003 | 60,79 | 14,58 | 1,5 | 0,00 |  | 90 | 160,09^*^ | 10,01^1^ | 150,08 | 375,58 | 1188 | 10 |
| N 859-2018 | 60,62 | 14,78 | 1,7 | 62,17 |  | 80 | 175,12^*^ | 10,76^1^ | 164,36 | 359,65 | 1229 | 4 |
| N 1797-2005 | 60,48 | 15,10 | 1,6 | 44,34 |  | 98 | 210,34 | 16,05 | 194,29 | 356,08 | 1248 | 3 |
| N 2642-1997 | 60,56 | 15,17 | 1,5 | 35,15 |  | 100 | 186,19^*^ | 10,27^1^ | 175,91 | 341,63 | 1344 | 2 |
| N 201-2011 | 60,22 | 16,47 | 1,7 | 10,90 |  | 85 | 202,15 | 4,53 | 197,62 | 268,88 | 1542 | 4 |
| N 16615-1997 | 60,35 | 16,27 | 1,9 | 24,00 | 1 | 90 | 226,11 | 5,55 | 220,56 | 289,08 | 1405 | 11 |
| N 14022-1997 | 60,41 | 16,00 | 1,7 | 8,14 |  | 85 | 226,28 | 23,75 | 202,53 | 295,36 | 1492 | 5 |
| N 855-2014 | 60,52 | 15,30 | 1,7 | 11,06 |  | 90 | 213,29 | 12,22 | 201,07 | 344,33 | 1366 | 1 |
| N 2298-2003 | 60,72 | 15,40 | 1,7 | 43,72 | 1 | 90 | 211,11 | 31,94 | 179,17 | 342,53 | 1391 | 4 |
| N 739-2011 | 60,75 | 16,14 | 1,6 | 49,91 |  | 80 | 237,87 | 12,81 | 225,06 | 303,93 | 1342 | 7 |
| N 1017-2017 | 60,70 | 15,99 | 1,8 | 17,05 |  | 50 | 215,41^*^ | 7,14^1^ | 208,27^1^ | 306,27 | 1365 | 7 |
| N 489-2011 | 60,72 | 15,76 | 2,2 | 19,19 | 1 | 60 | 210,86 | 17,36 | 193,51 | 307,65 | 1449 | 7 |
| N 5345-1996 | 61,58 | 15,81 | 1,8 | 5,70 | 1 | 80 | 236,63 | 21,27 | 215,36 | 335,27 | 1225 | 9 |
| N 391-2012 | 61,53 | 15,78 | 1,8 | 0,00 |  | 60 | 228,33 | 26,46 | 201,87 | 332,16 | 1287 | 16 |
| N 368-1992 | 61,29 | 15,76 | 1,9 | 10,70 | 1 | 80 | 218,70 | 19,93 | 198,76 | 324,43 | 1247 | 16 |
| N 1223-2008 | 60,95 | 15,51 | 1,5 | 0,00 | 1 | 90 | 204,64 | 13,82 | 190,82 | 327,01 | 1290 | 16 |
| N 780-2018 | 61,00 | 15,40 | 1,8 | 15,64 |  | 85 | 184,29^*^ | 11,69^1^ | 172,60^1^ | 321,53 | 1302 | 8 |
| N 9-1900 | 60,89 | 14,89 | 2,2 | 17,60 |  | 55 | 159,10 | 19,97 | 139,13 | 327,38 | 1362 | 8 |
| N 29-2010 | 60,84 | 15,07 | 1,9 | 9,34 |  | 75 | 182,24 | 16,66 | 165,58 | 337,25 | 1371 | 11 |
| N 1360-2009 | 60,76 | 15,19 | 1,6 | 10,40 |  | 95 | 207,47 | 30,34 | 177,13 | 350,75 | 1226 | 8 |
| N 989-2011 | 60,67 | 15,39 | 1,8 | 45,03 | 1 | 94 | 210,19 | 28,41 | 181,78 | 339,69 | 1332 | 3 |

^*^ Sites where we accounted for noise

Table S2. Our focal study species in our inventory, their organism group, substrate association and found frequency. Species that require highly specific habitats, such as very wet soils or calcareous substrates, were therefore not included in the survey. For epiphytic species it is specified if they occur on trees with high-pH bark properties or trees with low-pH bark.

| **Species** | **Organism group** | **Substrate** | **Frequency (nr sites)** |
| --- | --- | --- | --- |
| *Collema flaccidum* | Cyano lichen | Epiphytic (high-pH) | 1 |
| *Collema furfuraceum* | Cyano lichen | Epiphytic (high-pH) | 6 |
| *Collema spp.* | Cyano lichen | Epiphytic (high-pH) | 1 |
| *Collema subnigrescens* | Cyano lichen | Epiphytic (high-pH) | 7 |
| *Leptogium saturninum* | Cyano lichen | Epiphytic (high-pH) | 21 |
| *Leptogium teretiusculum* | Cyano lichen | Epiphytic (high-pH) | 2 |
| *Lobaria scrobiculata* | Cyano lichen | Epiphytic (high-pH), Epilithic | 1 |
| *Nephroma arcticum* | Cyano lichen | Epigeous | 6 |
| *Nephroma bellum* | Cyano lichen | Epiphytic (high-pH), Epilithic | 12 |
| *Nephroma parile* | Cyano lichen | Epiphytic (high-pH) | 18 |
| *Nephroma resupinatum* | Cyano lichen | Epiphytic (high-pH), Epilithic | 4 |
| *Parmeliella triptophylla* | Cyano lichen | Epiphytic (high-pH) | 23 |
| *Peltigera collina* | Cyano lichen | Epilithic, Epigeous | 3 |
| *Alectoria sarmentosa* | Cephalo/Chloro lichen | Epiphytic (low-pH) | 39 |
| *Arthonia leucopellea* | Cephalo/Chloro lichen | Epiphytic (low-pH) | 16 |
| *Bryoria bicolor* | Cephalo/Chloro lichen | Epiphytic (low-pH), Epilithic | 1 |
| *Bryoria nadvornikiana* | Cephalo/Chloro lichen | Epiphytic (low-pH) | 32 |
| *Calicium denigratum* | Cephalo/Chloro lichen | Epixylic | 4 |
| *Carbonicola anthracophila* | Cephalo/Chloro lichen | Epixylic | 6 |
| *Carbonicola myrmecina* | Cephalo/Chloro lichen | Epixylic | 7 |
| *Chaenotheca brachypoda* | Cephalo/Chloro lichen | Epixylic, Epiphytic | 10 |
| *Chaenotheca gracillima* | Cephalo/Chloro lichen | Epixylic | 3 |
| *Chaenotheca laevigata* | Cephalo/Chloro lichen | Epixylic | 2 |
| *Chaenotheca spp.* | Cephalo/Chloro lichen | Epiphytic | 1 |
| *Chaenotheca subroscida* | Cephalo/Chloro lichen | Epiphytic (low-pH) | 23 |
| *Chaenothecopsis fennica* | Cephalo/Chloro lichen | Epixylic (low-pH) | 2 |
| *Chaenothecopsis viridialba* | Cephalo/Chloro lichen | Epiphytic (low-pH) | 1 |
| *Cladonia parasitica* | Cephalo/Chloro lichen | Epixylic | 8 |
| *Hypogymnia bitteri* | Cephalo/Chloro lichen | Epiphytic (low-pH) | 2 |
| *Hypogymnia vittata* | Cephalo/Chloro lichen | Epiphytic (low-pH), Epilithic | 8 |
| *Icmadophila ericetorum* | Cephalo/Chloro lichen | Epixylic, Epigeous | 12 |
| *Lecidea botryosa* | Cephalo/Chloro lichen | Epixylic | 15 |
| *Letharia vulpina* | Cephalo/Chloro lichen | Epixylic | 1 |
| *Lobaria pulmonaria* | Cephalo/Chloro lichen | Epiphytic (high-pH), Epilithic | 13 |
| *Lopadium disciforme* | Cephalo/Chloro lichen | Epiphytic (low-pH) | 3 |
| *Microcalicium ahlneri* | Cephalo/Chloro lichen | Epixylic | 12 |
| *Ramalina sinensis* | Cephalo/Chloro lichen | Epiphytic (high-pH) | 1 |
| *Ramboldia elabens* | Cephalo/Chloro lichen | Epixylic | 5 |
| *Sclerophora coniophaea* | Cephalo/Chloro lichen | Epiphytic (low-pH) | 1 |
| *Anastrophyllum michauxii* | Bryophyte | Epixylic | 1 |
| *Anomodon viticulosus* | Bryophyte | Epiphytic (high-pH), Epilithic | 2 |
| *Buxbaumia viridis* | Bryophyte | Epixylic | 11 |
| *Calypogeia suecica* | Bryophyte | Epixylic | 2 |
| *Cephalozia affinis* | Bryophyte | Epixylic | 1 |
| *Crossocalyx hellerianus* | Bryophyte | Epixylic | 34 |
| *Dicranum flagellare* | Bryophyte | Epixylic | 2 |
| *Dicranum fragilifolium* | Bryophyte | Epixylic | 1 |
| *Geocalyx graveolens* | Bryophyte | Epixylic, Epigeous | 2 |
| *Herzogiella seligeri* | Bryophyte | Epixylic | 13 |
| *Herzogiella turfacea* | Bryophyte | Epixylic | 6 |
| *Homalia trichomanoides* | Bryophyte | Epiphytic, Epilithic | 3 |
| *Homalothecium sericeum* | Bryophyte | Epiphytic, Epilithic | 1 |
| *Hylocomiastrum pyrenaicum* | Bryophyte | Epilithic, Epigeous | 1 |
| *Hylocomiastrum umbratum* | Bryophyte | Epilithic, Epigeous | 11 |
| *Jungermannia leiantha* | Bryophyte | Epixylic, Epigeous | 2 |
| *Lophozia ascendens* | Bryophyte | Epixylic | 5 |
| *Lophozia longiflora* | Bryophyte | Epixylic | 14 |
| *Neckera complanata* | Bryophyte | Epiphytic, Epilithic | 4 |
| *Neckera crispa* | Bryophyte | Epiphytic (high-pH), Epilithic | 1 |
| *Neckera oligocarpa* | Bryophyte | Epilithic | 5 |
| *Neckera pennata* | Bryophyte | Epiphytic (high-pH) | 1 |
| *Nowellia curvifolia* | Bryophyte | Epixylic | 7 |
| *Odontoschisma denudatum* | Bryophyte | Epixylic | 1 |
| *Rhytidiadelphus subpinnatus* | Bryophyte | Epigeous | 5 |
| *Scapania apiculata* | Bryophyte | Epixylic | 1 |
| *Sphagnum quinquefarium* | Bryophyte | Epigeous | 25 |
| *Tritomaria exsecta* | Bryophyte | Epixylic | 1 |
| *Ulota crispa* | Bryophyte | Epiphytic (high-pH) | 3 |
| *Goodyera repens* | Vascular plant | Epigeous | 42 |
| *Lycopodium complanatum* | Vascular plant | Epigeous | 2 |
| *Moneses uniflora* | Vascular plant | Epigeous | 2 |
| *Monotropa hypopitys* | Vascular plant | Epigeous | 3 |
| *Neottia cordata* | Vascular plant | Epigeous | 7 |
| *Neottia ovata* | Vascular plant | Epigeous | 1 |
| *Pyrola chlorantha* | Vascular plant | Epigeous | 6 |

Table S3. (a) The range and mean of the climatic explanatory variables. (b) The Pearson correlation coefficients between the variables. Significance is indicated as follows: *** p < 0.001, ** p < 0.01, * p < 0.05, . p < 0.1.

| (a) |  |  | (b) | | | Correlation with the other variables | | | | |
| --- | --- | --- | --- | --- | --- | --- | --- | --- | --- | --- |
|  | Range | Mean | | SD |  | Summer precipitation (mm) | Distinct drought period (mm) | Prior/after distinct drought (mm) | Average summer precipitation | GDD |
| Drought intensity 2018 |  |  | |  |  |  |  |  |  |  |
| Summer precipitation (mm) | 143.5 – 265.2 | 206.3 | | 28.3 |  | 1 | 0.30* | 0.91*** | 0.22 . | -0.38** |
| Distinct drought period (mm) | 3.78 - 55.8 | 21.8 | | 12.0 |  | 0.30 * | 1 | -0.13 | 0.11 | -0.40** |
| Prior/after distinct drought (mm) | 120.8 – 245.3 | 184.5 | | 27.2 |  | 0.91*** | -0.13 | 1 | 0.18 | -0.22 . |
| Background climate |  |  | |  |  |  |  |  |  |  |
| Average summer precipitation (mm) | 262.0 – 448.1 | 327.2 | | 41.8 |  | 0.22. | 0.11 | 0.18 | 1 | -0.79*** |
| GDD (nr days over 10 years) | 814 – 1548 | 1291 | | 152 |  | -0.38** | 0.40** | 0.22. | -0.79*** | 1 |

Table S4. The standardized parameter estimates of model 2 on species richness at the site level, overall and categorized into organism group and substrate association, corresponding to the patterns shown in Fig. 3. Drought intensity is defined as the absolute precipitation (low precipitation is high drought intensity). In this model drought intensity is divided into two periods: the severely dry period 22^nd^ June – 27^th^ July, and the period before and after that. Negative coefficients thus denote a negative relationship between drought and species richness. Significance is indicated as follows: *** p < 0.001, ** p < 0.01, * p < 0.05, . p < 0.1. Non- and trend-significant coefficients are shown in light grey. The pseudo R^2^ variable is calculated for the main variables (excluding background climate).

|  | **Drought intensity** | | **Edge exposure** | **Interaction** | | **Background climate** | | **R^2^** |
| --- | --- | --- | --- | --- | --- | --- | --- | --- |
|  | During the extreme drought period | Prior and after | Edge exposure (%) | Extreme drought * Edge exp. | prior after * Edge exp. | Average summer precip, (mm) | Growing degree days | Pseudo R^2^ main variables |
| **Overall** ^1, P^ | -0.02 | -0.09 | -0.33 *** | -0.07 | -0.18 ** | -0.10 | -0.34 *** | 0.02 |
| **Organism group** |  |  |  |  |  |  |  |  |
| **Lichens**^1, P^ | -0.17 | -0.06 | -0.57 ** | -0.29 . | -0.30 ** | -0.08 | -0.54 ** | 0.20 |
| Cyanolichens^1, P^ | -0.19 | -0.14 | -0.64** | -0.06 | -0.36 ** | -0.07 | -0.35 . | 0.35 |
| Chlorolichens^1, P^ | -0.15 | 0.01 | -0.54 . | -0.27. | -0.46* | -0.08 | -0.70** | 0.06 |
| **Bryophytes**^1, P^ | 0.15 | -0.25 * | -0.15 | 0.11 | -0.06 | -0.16 | 0.1 | 0.30 |
| **Vascular plants**^2, QP^ | -0.07 | -0.11 | -0.15 | -0.28 . | -0.03 | -0.12 | 0.05 | 0.03 |
| **Substrate affiliation** |  |  |  |  |  |  |  |  |
| Epiphytic total^1, P^ | -0.06 | -0.03 | -0.46*** | -0.10 | 0.25** | -0.15 | -0.46*** | 0.13 |
| Epiphytic low-pH bark^2, P^ | 0.14 | 0.12 | -0.09 | -0.06 | -0.01 | -0.17. | -0.57 *** | 0 |
| Epiphytic high-pH bark^1,P^ | .0.15 | -0.09 | -0.49 * | 0.001 | 0.34 ** | -0.1 | -0.25 | 0.40 |
| Epilithic + epixylic^2, P^ | -0.001 | -0.12 | -0.22 | -0.05 | -0.22* | -0.15 | -0.29. | 0.21 |
| Epigeic^2, QP^ | -0.08 | -0.06 | -0.21 * | -0.20 . | 0.001 | -0.11 | -0.08 | 0 |

Table S5. The standardized parameter estimates from model 1 and model 2 on species richness at the subplot level within sites. Drought intensity is defined as the absolute precipitation (low precipitation is high drought intensity) during the different periods in the summer of 2018. Negative coefficients thus denote a negative relationship between drought and species richness. Edge exposure is a three-level ordinal factor with Interior, Weak edge effects and Strong edge effects, indicated by I, W, S respectively. Significance is indicated as follows: *** p < 0.001, ** p < 0.01, * p < 0.05, . p < 0.1. Non- and trend-significant coefficients are shown in light grey. We show the conditional pseudo R^2^ for the main variables (edge effects and drought severity).

|  | **Drought intensity** | | **Edge exposure** | **Interactions** | | **Background climate** | | **R^2^** |
| --- | --- | --- | --- | --- | --- | --- | --- | --- |
| **Model 1** | Summer of 2018 | | Edge exposure | Summer precipitation  * Edge exposure | | Average summer precip. (mm) | Growing degree days | Pseudo R^2^ main variables |
| Species richness within sites | -0.06 | | I: -6.3  W: -7.0  S: -6.5 | I: 0.06  W: 0.1  S: -0.03 . | | -0.25 | -0.66 *** | 0.46 |
| **Model 2** | Extreme drought period | Prior and after | Edge exposure | Extreme drought * Edge exp. | prior after * Edge exp. | Average summer precip, (mm) | Growing degree days | R^2^ main variables |
| Species richness within sites | -0.04 | 0.09 | I: -6.3  W: -7.1  S: -6.5 | I: -0.04  W: -0.19  S: -0.20 | I: 0.09  W: 0.20  S: 0.05 | -0.25 | -0.66** | 0.46 |

Table S6: Differences in coverage (and fecundity for *G. repens*) of the individual species between the different levels of edge exposure at the subplot level within woodland key habitats. Coverage of all species was log-transformed and the fecundity of *G. repens* arcsine transformed. We show the estimates between each edge effect category and their significance indicated as follows: p < 0.001, ** p < 0.01, * p < 0.05, . p < 0.1.

| **Organism group**  **Substrate affiliation** | **Species** | **Interior vs. strong edge** | **Interior vs. weak edge** | **Weak edge vs. strong edge** | **Conditional**  **Pseudo-R^2^** |
| --- | --- | --- | --- | --- | --- |
| Lichen (chloro)  Epiphytic | *B. nadvornikiana* | 1.57 . | 1.23 . | 0.34 | 0.49 |
| Lichen (chloro)  Epiphytic | *A. sarmentosa* | 1.53 * | 0.70 | 0.83 | 0.64 |
| Bryophyte  Epixylic | *C. hellerianus* | 3.68 *** | 2.47 *** | 1.21 | 0.38 |
| Vascular plant  Epigeous | *G. repens* Coverage  Fecundity | 5.63 ***  0.15 ** | 4.78 ***  0.13 *** | 0.85  0.015 | 0.67  0.002 |

**Supplementary Figures**


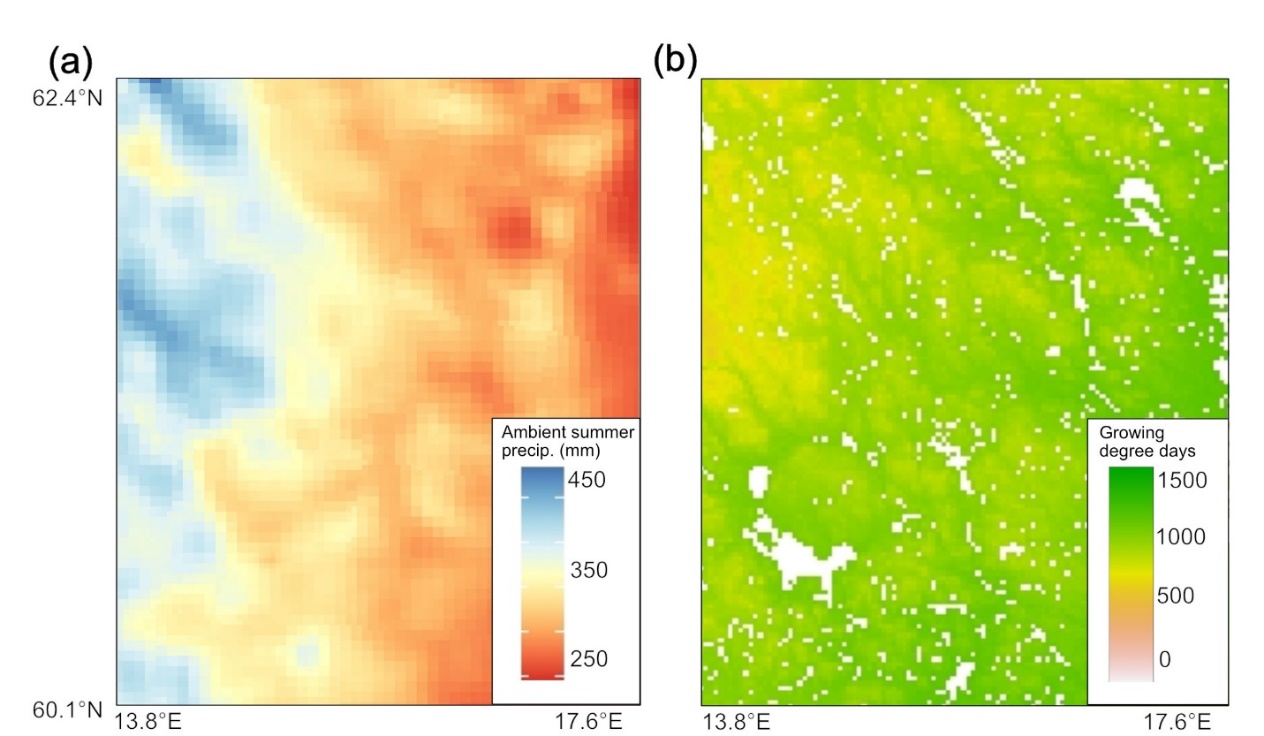


Fig. S1: The background macroclimate variables over our study area: (a) average summer precipitation over the period 2010-2017 and (b) growing degree days over the period 2000-2010


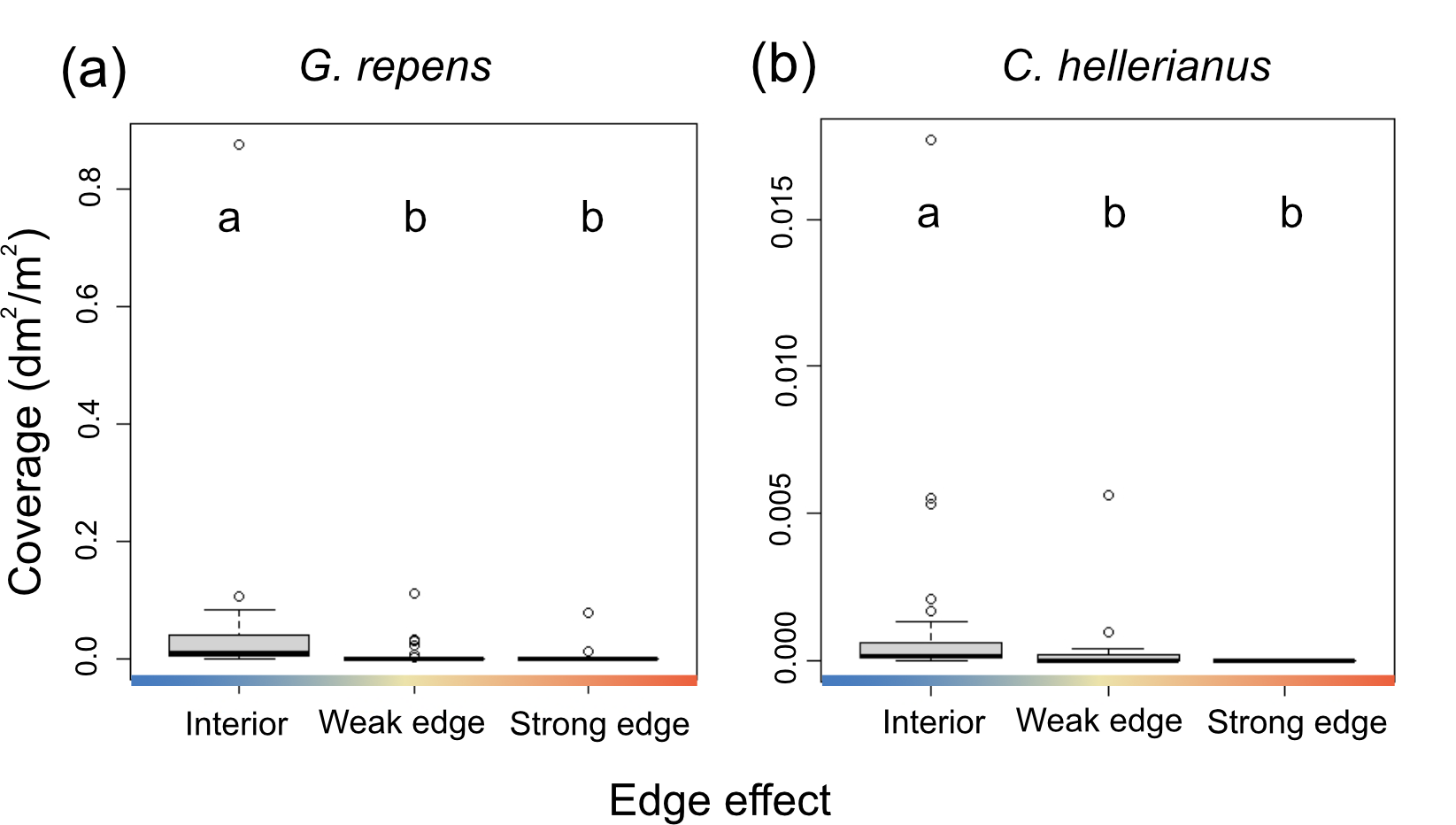


Fig. S2. Coverage of the orchid *Goodyera repens* (a) and the bryophyte *Crossocalyx hellerianus* (b) for different levels of edge exposure at the subplot level within woodland key habitats. The same boxplot as main Fig. 5, but including the outliers in the forest interior. Different letters above the boxplots (a and b) indicate statistical differences between the edge effects.


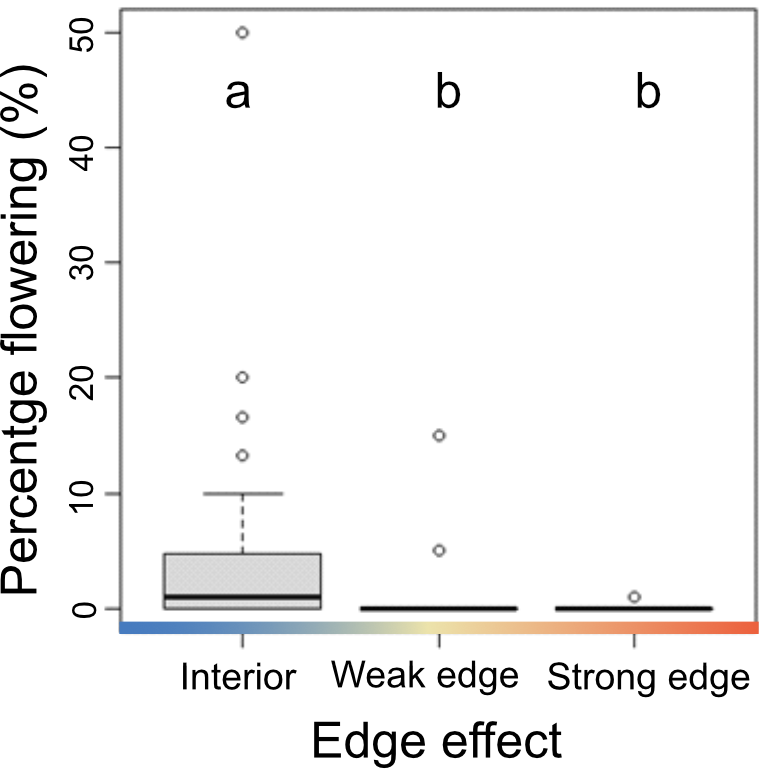


Fig. S4. Percentage of flowering individuals (as a measure of fertility) for the orchid *Goodyera repens* for different levels of edge exposure at the subplot level within woodland key habitats. Different letters above the boxplots (a and b) indicate statistical differences between the edge effects.

**
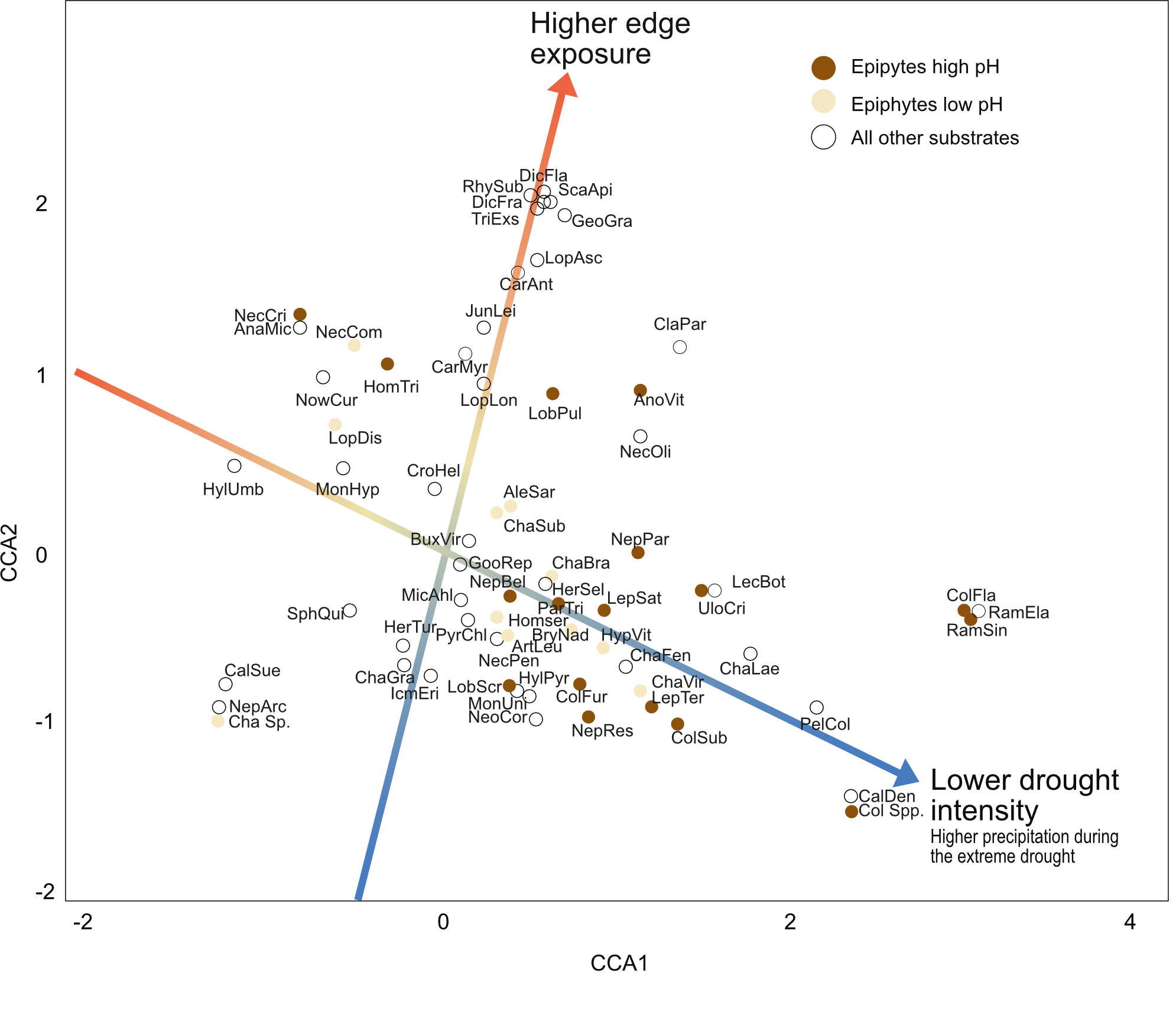
**

Fig. S5. The association between old-growth forest species composition and drought intensity and edge effects at the site level, showing the CCA species scores and the significant explanatory variables (p<0.05) after accounting for background climatic variables. It is the same CCA plot as Fig. 4. in the main text, but here the different colors represent species epiphytic on high-pH bark (dark brown) and species on low-pH bark (light brown). Species acronyms are based on the first three letters of the genus part and the first three letters of the species part of their scientific names, for example Goodyera repens = GooRep (full names can be found in Appendix Table S1). The eigenvalues were 0.37 for axis 1 and 0.30 for axis 2, and the inertia of the constrained (drought and edge exposure) and conditional (background climate) and variables were 0.12 and 0.14 respectively.

**Supplementary Methods**

**Statistical model specifications**

- **Model 1:** y ~ (Drought intensity summer 2018 × edge exposure) + Average summer precipitation + GDD
- **Model 2:** y ~ (Drought intensity extreme drought period × edge exposure) + (Drought intensity prior and after × edge exposure) + Average summer precipitation + GDD

Species richness

To test the hypothesis that drought intensity and edge exposure has reduced the number of understory species in the sites the year after the drought, we modeled species richness as a function of the explanatory variables as described above for model 1 and 2. To account for variation in size of the sites, we included the logarithmic of the area (in hectares) as an offset variable. We tested the association with drought intensity and edge exposure for the total number of species per site, as well as for number of species in the different categories based on organism group and substrate association. We merged epixylic and epilithic richness (on stone and logs), since they were few in each category and are likely to behave similarly (Hylander et al., 2005). In these models, we included an estimate of the number of downed logs as an additional co-variate. For the models that included epiphytic species associated with *Populus tremula* and/or *Salix caprea*, we excluded sites that did not contain these tree species, resulting in models with a sample size of 35 sites. We used generalized linear models assuming a Poisson distribution and accounted for over- and underdispersion when necessary, by using a quasi-Poisson distribution (Table 1). Overdispersion was tested for using the *AER* package (Kleiber & Zeileis, 2008) and underdispersion was determined based on the dispersion parameter in the summary output.

To investigate whether species richness at the subplot level was influenced by edge effects within sites, drought intensity and their interaction, we modeled species richness in each subplot according to model 1 and 2. Here edge exposure was a three-level ordinal factor with the previously defined strong edge effects, weak edge effects and forest interior for each subplot. We used nested generalized linear mixed effect models using the package *lme4* (Bates et al. 2015) assuming a Poisson distribution. We included site ID as a random effect and accounted for variation in subplot size (due to intersection with the woodland key habitat borders) by including it as an offset variable in logarithmic scale.

We checked the model diagnostics with the *plot* function for the models at the site level, and with the *DHARMA* package for the mixed models at the subplot level (Hartig 2020). We obtained the standardized model coefficients from the summary function and significance from an ANOVA. We calculated a conditional pseudo R-squared value for all models, but excluding the background climate covariables in order to assess how much of the variation was explained by drought intensity and edge effects. The pseudo R-squared for the models at the site level was obtained using the package *rsq*, using the *rsq.v* function which calculates the R-square based on the variance and is suitable for both Poisson and quasi-Poisson models (Zhang, 2017). The pseudo R-squared for the mixed effect models was obtained using the *r.squaredGLMM* function from the *MuMIn* package (Barton 2009). Note that the pseudo R-squared methods at the site level and at the subplot level are based on slightly different calculations and should not be compared with each other.

Community composition

To assess if the community composition of the focal species in the sites was associated with drought intensity and edge exposure, we conducted partial constrained correspondence analyses (CCA) with a species-by-site matrix as response variable, using the *vegan* package (Oksanen et al. 2013). Here we used the proportional coverage per site for each species ( area covered divided by size of the site). Again, we conducted the two previously described models. The background climatic variables (average summer precipitation and GDD) were included as conditioning variables, meaning they were accounted for in the models but their results were not shown. We simplified the models based on the lowest AIC using the step-function in *vegan*, extracted the marginal p-values based on 999 permutations, and plotted CCA axis 1 and 2 of the species scores overlain by the environmental variables. We analyzed the community composition for sites that had ≥ 5 focal species, to assure that species richness was not the main driver of differences in community composition between sites. Moreover, we only included sites where *Populus tremula* and/or *Salix caprea* were present. This resulted in a total of 32 sites included in this analysis.

Analyses of individual species

We examined how the four most common study species (*Goodyera repens, Alectoria sarmentosa*, *Bryoria nadvornikiana*, and *Crossocalyx hellerianus*) were related to the drought intensity in 2018, edge exposure, and their interaction. We assessed species responses both at the site level, as well as at the subplot level, again according to model 1 and 2..

For the site-level analysis, we first modeled presence/absence as a function of drought severity and edge exposure following model 1 and 2 for each species separately, using generalized linear models assuming a binomial distribution and including an offset variable to account for variation in size of the sites Second, we examined how the cover of each species was related to the drought and edge exposure, including only sites in which a species was present, using linear models. We log-transformed cover of all species in order to achieve normally distributed residuals. Finally, we modeled the proportion of fertile individuals of *G. repens* across sites as a function of the same explanatory variables, assuming beta distribution (+0.001 to avoid 0) using the package *betareg* (Grün et al., 2012).

At the subplot level, we assessed if the distribution of the four common species was influenced by drought severity and edge effects. Nested models that included each subplot separately were not possible to run, due to large zero inflation in the species coverage. For each site, we pooled subplots with strong edge effects, weak edge effects, and belonging to the forest interior, respectively, and calculated the proportional coverage of each species for each edge effect category, (species cover divided by the area of strong, weak and interior subplots, respectively). We modeled coverage following model 1 and 2, with edge exposure consisting of the three categories and site included as a random factor. Coverage for all species was log-transformed (plus 0.1 times the lowest coverage to avoid zero values) in order to meet the model assumptions. Due to poor model diagnostics, we also conducted several non-parametric tests for the coverage of *C. hellerianus*; a Kruskal Wallis test, a Friedman test from the package *agricolae* and a Wilcoxon rank test. All gave similar results to the linear model and we therefore proceeded by presenting results from this model. Lastly, we modeled the proportion of fertile individuals of *G. repens*, assuming a beta distribution with the function *glmmTMB*. Due to poor model diagnostics (encountered by the *Dharma* package) we also conducted linear models using an arcsine transformed fertility data.

**References**

Barton, K. (2009) Mu-MIn: Multi-model inference. R Package Version 0.12.2/r18. http://R-Forge.R-project.org/projects/mumin/

Bates, D., Mächler, M., Bolker, B., Walker, S., 2015. Fitting linear mixed-effects models using lme4. Journal of Statistical Software 67, 1–48. <https://doi.org/10.18637/jss.v067.i01>

Grün, B., Kosmidis, I., Zeileis, A., 2012. Extended beta regression in R: Shaken, stirred, mixed, and partitioned. Journal of Statistical Software*,*48.1–25. https://doi.org/[10.18637/jss.v048.i11](https://doi.org/10.18637/jss.v048.i11).

Hartig, F., 2020. DHARMa: Residual diagnostics for hierarchical (multi-level / mixed) regression models. R package version 0.3.3.0. <http://florianhartig.github.io/DHARMa/>

Hylander, K., Dynesius, M., Jonsson, B.G., Nilsson, C., 2005. Substrate Form Determines the Fate of Bryophytes in Riparian Buffer Strips. Ecological Applications 15, 674–688. <https://doi.org/10.1890/04-0570>

Kleiber, C., Zeileis, A., (2008). Applied Econometrics with R*.* Springer-Verlag, New York. ISBN 978-0-387-77316-2. [https://CRAN.R-project.org/package=AER](https://cran.r-project.org/package=AER).

Oksanen, J., Blanchet, F.G., Kindt, R., Legendre, P., Minchin, P.R., O'Hara, R.B., Simpson, G.L., Solymos, P., Stevens, M.H.H., Wagner, H., 2013. Vegan: Community Ecology Package, Version 2.0–8.

Zhang, D., 2017. A coefficient of determination for generalized linear models. The American Statistician 71, 310–316. <https://doi.org/10.1080/00031305.2016.1256839>
